# Spatial distance between sites of sampling associated with genetic variation among *Neospora caninum* in aborted bovine foetuses from northern Italy

**DOI:** 10.1186/s13071-020-04557-6

**Published:** 2021-01-13

**Authors:** Luca Villa, Pavlo Maksimov, Christine Luttermann, Mareen Tuschy, Alessia L. Gazzonis, Sergio A. Zanzani, Michele Mortarino, Franz J. Conraths, Maria Teresa Manfredi, Gereon Schares

**Affiliations:** 1 Department of Veterinary Medicine, Università degli Studi di Milano, Via dell’Università 6, 26900 Lodi, Italy; 2 Friedrich-Loeffler-Institut, Federal Research Institute for Animal Health, Institute of Epidemiology, Südufer 10, 17493 Greifswald-Insel Riems, Germany; 3 Friedrich-Loeffler-Institut, Federal Research Institute for Animal Health, Institute for Immunology, Südufer 10, 17493 Greifswald-Insel Riems, Germany

**Keywords:** Neosporosis, Microsatellite typing, Multilocus genotyping, Bovine abortion, Holstein friesian cattle, Italy

## Abstract

**Background:**

*Neospora caninum*, a coccidian protozoan, represents an important cause of bovine abortion. Available *N. caninum* strains show considerable variation *in vitro* and *in vivo*, including different virulence in cattle. To which extent sexual recombination, which is possible in the intestines of domestic dogs and closely related carnivores as definitive hosts, contributes to this variation is not clear yet.

**Methods:**

Aborted bovine foetuses were collected between 2015 and early 2019 from Italian Holstein Friesian dairy herds suffering from reproductive problems. A total of 198 samples were collected from 165 intensive farms located in Lombardy, northern Italy. *N. caninum* samples were subjected to multilocus-microsatellite genotyping using ten previously established microsatellite markers. In addition to our own data, those from a recent study providing data on five markers from other northern Italian regions were included and analysed.

**Results:**

Of the 55 samples finally subjected to genotyping, 35 were typed at all or 9 out of 10 loci and their individual multilocus-microsatellite genotype (MLMG) determined. Linear regression revealed a statistically significant association between the spatial distance of the sampling sites with the genetic distance of *N. caninum* MLMGs (*P* < 0.001). Including data from this and a previous North Italian study into eBURST analysis revealed that several of *N. caninum* MLMGs from northern Italy separate into four groups; most of the samples from Lombardy clustered in one of these groups. Principle component analysis revealed similar clusters and confirmed MLMG groups identified by eBURST. Variations observed between MLMGs were not equally distributed over all loci, but predominantly observed in MS7, MS6A, or MS10.

**Conclusions:**

Our findings confirm the concept of local *N. caninum* subpopulations. The geographic distance of sampling was associated with the genetic distance as determined by microsatellite typing. Results suggest that multi-parental recombination in *N. caninum* is a rare event, but does not exclude uniparental mating. More comprehensive studies on microsatellites in *N. caninum* and related species like *Toxoplasma gondii* should be undertaken, not only to improve genotyping capabilities, but also to understand possible functions of these regions in the genomes of these parasites.
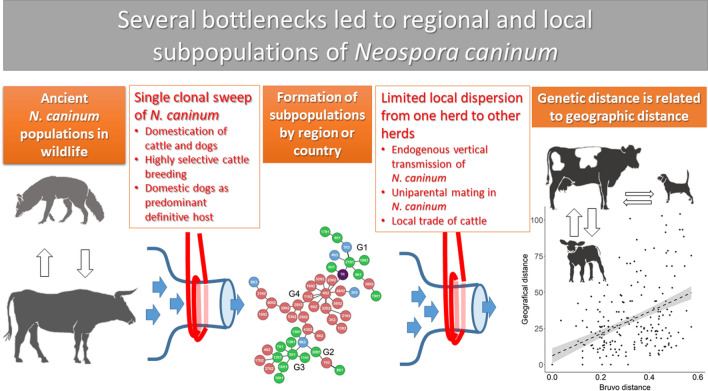

## Background

*Neospora caninum* is a protozoan coccidian parasite closely related to *Toxoplasma gondii* and *Besnoitia besnoiti*. It has a worldwide distribution and causes foetal losses or stillbirth in livestock, especially cattle [1,2]. Domestic dogs and other phylogenetically closely related wild carnivores like coyotes, wolves, or dingoes are the only known definitive hosts of *N. caninum* [1,3–5], i.e. hosts, in which sexual recombination can take place. The main mode of transmission in cattle, which are important intermediate hosts of the parasite, seems to be endogenous vertical transmission [1,2], i.e. transmission from a persistently or chronically infected dam to her offspring [6]. Exogenous vertical transmission to a foetus or an unborn calf occurs in cattle that became infected during pregnancy after the ingestion of oocysts shed by a definitive host [6,7]. Transmission via oocysts by definitive hosts, most likely farm dogs, seems to occur frequently in cattle populations. These infections are the likely cause of epidemic abortions in cattle herds [8]. Such abortion storms were reported to be associated with both (i) low avidity, i.e. a recently established antibody response against *N. caninum* [8,9], and (ii) an identical microsatellite pattern in *N. caninum* detected in several foetuses of a single herd [10].

A recent study on the genomes of 50 *N. caninum* isolates collected worldwide from a wide range of hosts using 19 linked and unlinked genetic markers showed that there is only a single genotype of *N. caninum* worldwide [11]. Moreover, whole genome sequencing of seven isolates from two continents revealed less than 10^4^ bi-allelic single nucleotide polymorphisms (SNPs), which is very little compared to the situation in the genome of the closely related apicomplexan parasite *T. gondii* with > 10^6^ SNPs between the compared strains [11]. Since > 50% of the SNP clustered in six haploblocks which had been already partially observed in a former study [12], it was concluded that uni-parental reproduction together with a non-sexual expansion formed the actual worldwide *N. caninum* population rather than non-sexual expansion alone [11]. The extremely limited genetic diversity was explained by genetic bottlenecks during the domestication of cattle in the Near East probably about 10,000 years ago [11]. Breeding and moving specific cattle breeds including the Holstein Friesian breed examined here during the following centuries may also have contributed [13]. The domestication of dogs may have formed another bottleneck, which shaped the *N. caninum* population that exists today [11].

Nevertheless, *N. caninum* is less uniform than the previously mentioned study [11] suggests. In fact, different *N. caninum* isolates show large differences *in vitro* [14–16] and *in vivo* [17–19], also in cattle [20–23]. The pioneering work of Regidor-Cerrillo et al. [24] contributed a number of microsatellite markers that allow fingerprinting *N. caninum* isolates or DNAs and undertake population studies.

We aimed at genotyping *N. caninum* in aborted bovine foetuses from Lombardy, one of the most important dairy cattle production areas in Italy [25]. In this region, where the Italian Holstein Friesian breed prevails, *N. caninum* was suspected as an important cause of abortion (MTM, unpublished data). Moreover, a high *N. caninum* seroprevalence was revealed in cattle from northern Italy [26,27]. We therefore determined the proportion of *N. caninum* PCR-positive aborted foetuses in this area and characterised the available isolates by multi-locus microsatellite genotyping.

A previous worldwide study [28], a South-American study [29], a local study from Spain [30], and a recent study conducted in northern Italy [31] suggested the existence of *N. caninum* sub-populations specific for particular countries or regions. The North-Italian study conducted predominantly in two areas (Piedmont and Veneto-Trento) close to our study area suggested at least three sub-populations. One aim was to find out how *N. caninum* isolates in aborted foetuses from Lombardy fit into the pattern of these sub-populations. To understand the reasons for genetic differences among *N. caninum* of one region, we used detailed geographic information on the sites, where *N. caninum*-positive foetal material had been sampled to assess, whether genetic differences were associated with the spatial distances of farms. A third aim was to look at farms with repeated submission of *N. caninum*-positive foetuses and to investigate, if the microsatellite data would support either uni-parental or multi-parental sexual reproduction.

## Material and methods

### Study area

**Fig. 1:**
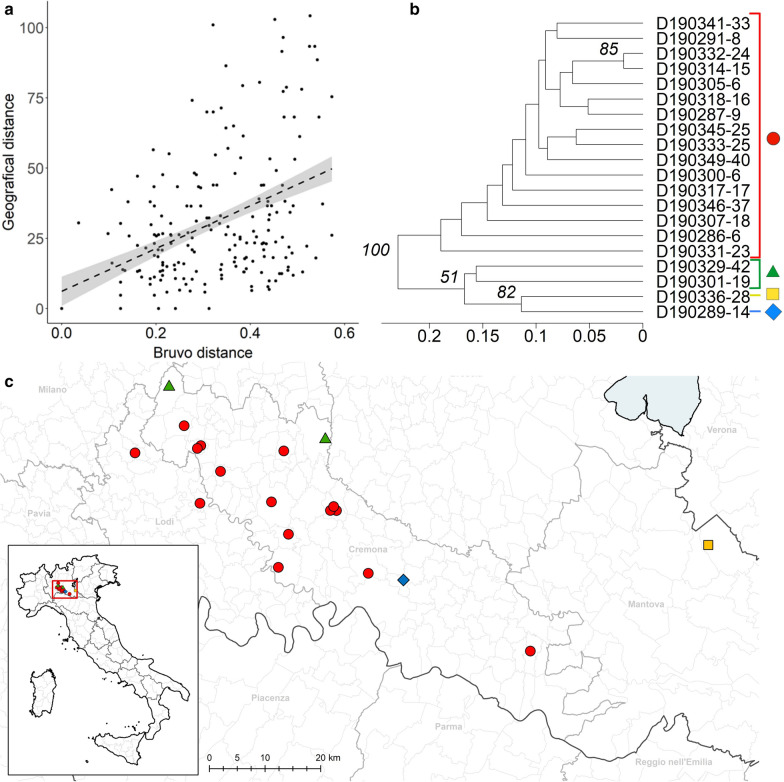
Genetic distance between *N. caninum* isolates from bovine foetuses collected in Lombardy, northern Italy, is associated with the geographical distance of the sampling sites. **a** Graphical representation of a linear regression model to associate Bruvo’s genetic distance of *N. caninum* in bovine foetuses and spatial distance of sampling sites (km). **b** Dendrogram representing the results of K-means clustering using Bruvo’s distance with non-parametric bootstrapping; the analysis identified at least four groups (different colours of sample designation, consisting of a string of sample ID, village name, and farm number), separated by bootstrap values > 50. **c** Map showing farm locations, using the same colours as for the groups in the dendrogram

All farms, from which aborted foetuses had been sampled, were located in the “Bassa Padana” in the Po valley. This area comprises the territory of the provinces of Lodi, Cremona, Mantova, Pavia, and the south of the provinces of Milano and Brescia (Fig. 1). This is one of the largest dairy production areas in Italy and stands out for the high density of cattle farms, mainly based on Italian Holstein Friesian under the intensive production system. In particular, the Italian National Zootechnical Registry counted 5446 dairy farms hosting 1,071,164 animals in the Lombardy region, corresponding to 20.7% and 40.8% of all farms and animals in Italy, with an annual production of 5,215,408 tons of milk (http://www.assolatte.it/zpublish/4/uploads/4/news_down/15641362883148578112_RAPPORTO%20ASSOLATTE%202018.pdf; last access 06.08.2020). Most farms in the area host between 100 and 500 animals, but several farms hold more than 500 cattle (National Zootechnical Database, https://www.vetinfo.sanita.it; last access 06.08.2020). In these dairy cattle farms, mainly animals born in the farm are used for replacement.

The sites have an average altitude of about 69 m above sea level. The climate is continental, typical of the Po valley, with hot muggy summers with a few thunderstorms and cold and foggy winters with some snow. There is a large annual thermal excursion with a mean maximum temperature of 25–28 °C and a mean minimum temperature of − 1 to – 2 °C. Rainfall is distributed over the year with peaks in spring and autumn. The average total annual rainfall ranges between 700 and 1200 mm.

### Sample collection

Aborted bovine foetuses were collected between 2015 and early 2019 from Italian Holstein Friesian dairy herds suffering from reproductive problems; previous unpublished diagnostic results (i.e. by serology or PCR) had suggested endemic *N. caninum* associated abortion problems in these herds. Overall, 198 samples were collected from 165 intensive farms located in the Po valley (Provinces of Cremona, Lodi, Mantova, and Milano). Abortion events had been notified by the farm veterinarian, foetuses were maintained refrigerated and collected directly from the farms or submitted to the laboratory. In the laboratory, a pool of organs was prepared from each aborted foetus, which included brain, lung, and liver. Pools were used to streamline the diagnostic procedure and to keep costs at a minimum. Target organs, i.e. brain, lung, and liver, were isolated from aborted foetuses, and about 10 g of each organ was pooled into a 50-ml tube and subsequently homogenized mechanically after the addition of 10 ml of 1 × phosphate buffered saline using a laboratory homogenizer (Omni GLH 850, OMNI International, Kennesaw, GA, USA) with Soft Tissue Omni Tips Plastic Probes (Omni GLH 850, OMNI International, Kennesaw, GA, USA) to avoid cross-contamination. Homogenized pools were stored at – 20 °C until DNA extraction. The geographical localization (municipality and province as well as the geographic coordinates) of each farm with abortion episodes was recorded along with the data for each aborted foetus.

### DNA extraction

Genomic DNA was extracted from tissue homogenates of aborted foetuses using a commercial kit (NucleoSpin^®^ Tissue, Macherey-Nagel, Düren, Germany), according to the manufacturer’s instructions. To optimize DNA extraction from aborted foetuses, a 200 µl aliquot of the homogenate was pre-lysed with buffer T1 and proteinase K as recommended for the NucleoSpin Tissue Kit, by scaling up eight times the volumes used for the initial digestion. After digestion (56 °C, overnight), 230 µl of the final suspension (1840 µl) was taken and the protocol of the NucleoSpin Tissue kit followed as recommended. A negative extraction control was included in each batch of ~ 24 samples, which was subsequently tested by real-time PCR in the same way as the samples from aborted foetuses were analysed.

### Screening *N. caninum* real-time PCR

Foetal DNA samples were initially subjected to a *N. caninum*-specific real-time PCR with the primers and probes targeting the Nc5 gene as previously described [32,33]. Primers were used at a final concentration of 600 nM and the fluorogenic probe (NeoProbe) at 100 nM. Ten μl of 2 × iQ Supermix (Bio-Rad Laboratories GmbH, Feldkirchen, Germany) was added to each reaction. For PCR analysis, 5 μl of DNA from each sample was used in a 20 μl reaction on a 96-well reaction plate. The thermal cycling protocol consisted of an initial polymerase activation and DNA denaturation at 95 °C for 5 min, followed by 46 cycles of amplification including denaturation at 95 °C for 10 s and annealing/extension at 58 °C for 30 s. NC-1 [34] was included as a positive control, while water PCR Reagent (Sigma-Aldrich) was used as a negative control; negative extraction but no endogenous controls were included.

### Microsatellite typing

Samples confirmed as positive for *N. caninum* were subjected to multilocus-microsatellite typing using ten microsatellite markers (MS1B, 2, 3, 5, 6A, 6B, 7, 10, 12, and 21). Nested-PCR (n-PCR) techniques were used for the amplification of microsatellite-containing regions from *N. caninum* DNA from aborted foetuses. Microsatellites MS2 and MS10 were amplified and sequenced using previously described primers and protocols [35]. The microsatellites MS1B, 3, 5, 6A, 6B, 7, 12, and 21 were analysed by nested-PCR and fragment lengths determined by capillary electrophoresis [10]. Forward primers of the internal PCRs were labelled with either 6-FAM, HEX, or NED dyes to allow the simultaneous length determination of three different microsatellites per capillary electrophoresis. In addition, previously sequenced microsatellite-containing fragments from the *in vitro*-grown *N. caninum* NC-1 strain [36] were included in the amplifications, the sequencing, and sizing analyses as a positive control.

For all n-PCRs, primers were used at a final concentration of 0.5 µM and dNTPs at 250 µM each (Amershan Biosciences, Piscataway, NJ, USA). DyNAzyme II DNA polymerase (Finnzymes, Espoo, Finland) was added at 1 U/25 µl with the provided buffer. The reaction mix was supplemented with bovine serum albumin at a concentration of 20 µg/ml; 1.5 µl of genomic DNA or 1 µl of amplification product was used as template for the external and internal PCR amplification steps, respectively. Water PCR Reagent (Sigma-Aldrich) was used as a negative control. DNA from cell culture-derived *N. caninum* tachyzoites (NC-1) was used as a positive control. The PCR reactions were performed in a thermal cycler (Eppendorf Mastercycler, Personal Thermal Cycler). The external PCR was performed with an initial denaturation step of 94 °C for 5 min, followed by 35 cycles of denaturation (1 min at 94 °C), annealing (1 min at 60 °C for all MS, except for MS2: 50 °C, and MS10: 54 °C) and extension (1 min at 72 °C), and a final extension step at 72 °C for 10 min. The internal PCR consisted of an initial denaturation of 94 °C for 5 min, followed by 35 cycles of denaturation: 1 min at 94 °C, annealing: 1 min at 60 °C (MS2, 10) or 30 s at 52 °C (MS1B, 3, 5, 6A, 21), 54 °C (MS6B, 12) or 60 °C (MS7) and extension: 1 min at 72 °C (MS2, 10), 30 s at 70 °C (MS1B, 3, 5, 6A, 6B, 12, 21) or 65 °C (MS7), and a final extension step at 72 ° C (MS2,10), 70 °C (MS1B, 3, 5, 6A, 6B, 12, 21) or 65 °C (MS7) for 10 min.

The amplification products were visualized after electrophoresis in 2% agarose gels stained with ethidium bromide using a 100 bp DNA ladder (Invitrogen GmbH, Karlsruhe, Germany) as a size standard.

MS2 and MS10 were Sanger-sequenced because these microsatellite markers consist of three sub-loci (or motifs) each. This situation could only be resolved by sequencing and not by capillary electrophoresis. For sequencing MS2 and MS10, bands of the expected size were excised from the agarose gels and purified with a commercial kit (NucleoSpin^®^ Gel and PCR Clean-up, Macherey-Nagel, Düren, Germany), following the manufacturer’s instructions. Purified amplification products were then cloned into a commercially available vector (pGEM^®^-T Easy Vector System I, Promega, Mannheim, Germany) and used to transform chemically competent *Escherichia coli* (OneShot TOP10, Thermo Fisher Scientific, Langenselbold, Germany). The transformed *E. coli* were cultivated, and the plasmid DNA was subsequently collected using a commercial kit (QIAprep Spin Miniprep Kit, Qiagen, Hilden, Germany), according to the manufacturer’s instructions. Sequencing was finally performed using the BigDye Terminator v1.1 Cycle Seq. Kit (Thermo Fisher Scientific, Langenselbold, Germany) and passage with NucleoSEQ Columns (Macherey-Nagel, Düren, Germany) for cleaning up nucleic acids, in an ABI 3130 capillary sequencer (Thermo Fisher Scientific, Langenselbold, Germany).

For all other MS, *N. caninum*-positive samples were analysed on an ABI 3130 capillary sequencer (Thermo Fisher Scientific, Langenselbold, Germany). A ROX dye-labelled standard (GeneScan™ 500 ROX™ dye Size Standard, Thermo Fisher Scientific, Langenselbold, Germany) was included in each analysis as a size reference. The results were analysed using the Geneious 1.11.5 software (Biomatters, Inc., 2365 Northside Dr., Suite 560, San Diego, CA 92108, USA).

### Statistical analysis

Differences in *N. caninum*-specific real-time PCR results (Ct values) in relation to the number of microsatellite markers typed were assessed using the command “wilcox.test” from the package “stats” in R, version 3.5.3 (R Foundation for Statistical Computing, Vienna, Austria; http://www.R-project.org). Microsatellite marker-specific differences in the number of repeats between *N. caninum* isolates from different regions were assessed using the command “pairwise.wilcox.test” from the package “stats” in R, version 3.5.3 “BH adjusted” option because of multiple testing [37].

For calculating the relative genetic distance between microsatellite genotypes, we used a method that specifically permits analysing organisms with different polyploidy levels and takes stepwise mutational processes into account [38]. Pairwise Bruvo’s genetic distance was calculated using microsatellite data of all DNA samples from aborted foetuses in Lombardy, for which a complete MLMG was obtained (*n* = 20). Since MS10 and MS2 were combined microsatellite markers, which consist of three sub-loci (or motifs) each, data on these sub-loci were included separated into the data set. Calculations were done by R, version 3.5.3., employing “Poppr”, version 2, a R package designed for the analysis of populations with mixed modes of reproduction [39], by using the command “bruvo.dist”.

To visualize the relatedness of individual MLMGs, a dendrogram was established using the “Poppr” package representing the results of K-means clustering using Bruvo’s distance with non-parametric bootstrapping, a process that consists of randomly sampling loci with replacement, recalculating the dendrogram, and grossing up the bootstrap support (in percent of success).

For testing of a standardized index of association ($I_{{\text{A}}}^{{\text{S}}}$) by multilocus linkage disequilibrium (LD) among different genotypes including nine loci the LIAN v 3.7 web interface program (http://guanine.evolbio.mpg.de/cgi-bin/lian/lian.cgi.pl/query) was applied as described [28,40].

Testing population differentiation was done by likelihood ratio G‐statistic [41], using the function “test.between” from the R-package “HierFstat” [42].

Geographic distance based on the geographic coordinates of the farms were calculated (straight-line distance, accounting for curvature of the earth) using the command “spDists” of the package “sp”, version 1.4-2, in R. To determine the relatedness of Bruvo’s distance, geographic distance and the time between samplings linear regression was performed using the “lm” command in R, version 3.5.3 from the package “stats”.

The eBURST software [43] was used to generate networks based on MLMGs using the double-locus option (DVL—at least 7 shared loci of 9). Samples with missing data were excluded from the analysis. As the eBURST analysis was performed on samples from this study and those from a previous study, the analysis was restricted to those microsatellite loci that were used in both studies. Since MS10 is a combined microsatellite marker, which consists of three sub-loci (or motifs), these sub-loci were analysed separately. The same data (MLMGs data collected in the present study on foetuses from Lombardy and data from a previous study) were also analysed by a Principal Coordinates Analysis (PCoA) using the command “dudi.pco” employing the R package “Poppr”, version 2.

Figures were assembled using R, version 3.5.3 or 4.0.0 (packages “ggplot2”, “reshape” and “scales”).

## Results

### Overall *N. caninum* DNA findings in aborted foetuses

Out of 198 aborted foetuses 55 were positive for *N. caninum* by real-time PCR, yielding a prevalence of 27.8% (presence of the parasite in pooled tissue homogenates of brain, lung, and liver); 43 farms recorded at least one positive foetus (26.1%). Overall, 55 samples from 43 herds of foetuses collected were subjected to microsatellite typing.

### Relationship between real-time PCR results and success in genotyping

The majority of the 55 DNA samples subjected to multilocus microsatellite typing were typed at all 10 (*n* = 20) or at 9 (*n* = 15) loci. Nine DNA samples could be typed at eight (*n* = 2), seven (*n* = 2) or six (*n* = 6) loci. The remaining ten DNA samples could only be typed at five (*n* = 3), four (*n* = 3), three (*n* = 2), two (*n* = 1), or one (*n* = 1) of the loci.

The numbers of genotyped loci were related to the amount of parasitic DNA in the sample as reflected by the Ct values determined by real-time PCR (Table 1).

**Table 1:** *N. caninum* DNA content as reflected by the Ct value in samples of aborted foetuses and success in genotyping

Typing success, no. of loci typed	Ct value, mean ± standard deviation	Minimum–maximum Ct	*P* value, Wilcoxon rank sum test to assess difference in Ct values
10 or 9	30.4 ± 3.2	26.2–42.4	Reference
8–1	33.0 ± 1.8	29.2–36.5	*P* = 0.00013

### Microsatellite typing on individual farms at different time points revealed differences in the MLMGs

In seven farms, more than one foetus had been sampled and detected *N. caninum* DNA typed in the study period from 2015 to 2018. Most of the differences in typing comprised only one or two repeat units in individual microsatellites (Table 2). However, in five farms, more prominent differences per locus (i.e. differences of 3 or even more repeat units per locus) were observed. In herd 6, i.e. the farm with the largest number of foetuses analysed, 48.1% of the comparisons of individual loci revealed differences (Tables 2, 3). These more prominent differences affected the loci MS7 and MS21 (chromosome 7a), MS10 (MS10.2, chromosome 8), and MS6A (chromosome 10) (Table 3).

**Table 2:** Comparison of the MLMG patterns in dairy cattle farms in northern Italy, where more than one *Neospora caninum*-positive foetus had been sampled and genotyped during the study period

Farm no.	Number of foetuses per farm (period)	Number of pairs of loci or sub-loci with differences of…				Differences/total number of comparisons (%)
		1 Repeat	2 Repeats	3 Repeats	> 3 Repeats	
4	2 (2015)	4	–	–	–	3/14 (21.4)
6^a^	4 (2015–16)	30	6	3	–	39/81 (48.1)
9	2 (2015)	1	1	–	–	2/3 (66.7)
10^a^	2 (2016)	2	–	–	1	3/10 (30.0)
15^a^	2 (2017)	2	–	–	1	3/11 (27.3)
17^a^	3 (2017–18)	4	–	–	2	6/13 (46.2)
25	2 (2017–18)	2	1	–	–	3/14 (21.4)
35^a^	2 (2018)	1	–	–	1	2/5 (40.0)

^a^Table 3 provides details on microsatellite typing results for farms, where loci with 3 or > 3 repeats were observed

**Table 3:** MLMG patterns on dairy cattle farms from northern Italy where more than one *Neospora caninum-*positive foetus had been sampled during the study period, which showed differences (bold numbers) in length for ≥ 3 repeats at microsatellite loci on various chromosomes (Chr2, 7a, 8, 9, 10, 12)

Farm	Date	Chr 2	Chr 7a			Chr 8	Chr 9	Chr 10		Chr 12	
		MS2, *x*-*y*-*z*, *(AT)x*-TTGTATC-*(AT)y*-GT-*(AT)z*	MS1B, *x *= *y* + *z*, *(AT)y*-AC-*(AT)z*	MS7, *x*, AT-AA-*(TA)*_*X*_-GG	MS21, *x *= *y*+4, TG-*(TACA)*_3_-TACC-*(TACA)*_*y*_TT	MS10, *x*-*y*-*z*, AGT-*(ACT)*_*X*_-*(AGA)*_*Y*_-*(TGA)*_*Z*_-CAA	MS5, *x*, CG-*(TA)*_*X*_-TGTAGG	MS6A, *x*, GC-*(TA)*_*X*_-AC	MS6B, *x*, CC-*(AT)*_*X*_-GT	MS12, *x*, GC-*(GT)*_*X*_-GC	MS3, *x*, GC*-(AT)*_x_-AA
6	May 2015	6-8-2	12	15	6	6-23-10	11	**22**	13	17	13
	February 2016	7-10-2	12	15	6	6-25-10	11	**25**	13	16	12
	April 2016	6-11-2	13	13	NA	5-25-10	11	**20**	14	16	11
	June 2016	6-11-2	12	14	6	6-24-9	11	**22**	13	16	12
10	January 2016	5-10-2	12	15	**3**	NA	11	21	13	16	12
	April 2016	6-11-2	12	15	**6**	6-27-10	11	21	13	NA	12
15	August 2017	6-11-2	12	15	6	6-**19**-10	NA	NA	NA	16	12
	August 2017	6-11-2	12	14	6	6-**28**-9	11	18	13	16	12
17	May 2017	6-10-2	NA	**14**	6	NA	11	NA	NA	17	12
	November 2017	6-9-2	12	**15**	6	7-24-12	11	**21**	13	16	12
	February 2018	NA	12	**10**	6	NA	NA	**13**	12	NA	NA
35	July 2018	6-11-2	12	15	6	NA	11	**19**	13	16	12
	October 2018	NA	NA	15	6	NA	12	**13**	NA	NA	12

### Relationship between the spatial distance of sampling sites and the genetic distance of *N. caninum* isolates

For 20 of the *N. caninum*-positive samples of bovine foetuses from Lombardy, which had been typed at the complete set of microsatellite loci (*n* = 10, this study), pairwise Bruvo’s genetic distances were calculated. Bruvo’s genetic distance and the geographical distance between sampling sites of the individual *N. caninum*-positive foetuses (i.e. based on the geographical coordinates of 17 farms) were tested for correlation. Linear regression revealed that Bruvo’s genetic distance correlated statistically significantly with the geographical distance between the sampling sites (Fig. 1a; Table 4). The model (Model 1, Table 4) had an adjusted *R*^2^ of 19.5%. Including the number of days between the sampling of foetuses into the model did not improve the model significantly (Model 2, Table 4).

**Table 4:** Linear regression models to characterize the association between genetic distance (Bruvos’s genetic distance) of *N. caninum* isolates and the spatial distance of the sampling sites or the time between dates of sampling

Model (adjusted *R*^2^)	Variable	Estimate	Standard error	*T* value	Pr (> |*t*|)
1 (19.5%)	Intercept	0.2378241	0.0100212	23.732	< 2e−16***
	Geographical distance (km)	0.0025708	0.0002615	9.831	< 2e−16***
2 (19.8%)	Intercept	0.2502685	0.0143581	17.43	< 2e−16***
	Geographical distance (km)	0.0026139	0.0002638	9.91	< 2e−16***
	Time between dates of sampling	0.0013088	0.0010820	− 1.21	0.227

^***^P < 0.001

Bruvo’s genetic distance with non-parametric bootstrapping allowed the identification of at least four groups, separated by bootstrap values > 50 (Fig. 1b). The farms, from which the largest group of samples originated (Fig. 1b, red), were located with two exceptions central-north in the Cremona district (Fig. 1c, red). The farms, from which the remaining samples had been derived, were located in the far north (Fig. 1c, green), in the south of the Cremona district (Fig. 1c, blue), or in the neighbouring district of Mantova (Fig. 1c, yellow).

### An eBURST and PCoA analysis revealed different groups or clusters of *N. caninum* MLMGs in northern Italy

In addition to the microsatellite data established in this study (Lombardy, *n* = 25), further data on 50 *N. caninum* isolates from northern Italy covering mainly other regions than Lombardy (Piedmont, *n* = 17; Veneto-Trento, *n* = 6, including also one goat isolate), Lombardy (*n* = 1), and a further North-Italian bovine *N. caninum* isolate (*n* = 1) were analysed. These additional data were available from a recent study [31]. Since the latter study had employed a set of microsatellite markers that overlapped only partially with the one we used, our analysis was restricted to MS6A, MS6B, MS10, MS12, and MS21, for which data were available from the present and the previous study. Linkage disequilibrium (LD) was assessed for the entire population excluding the goat sample; results ($I_{{\text{A}}}^{{\text{S}}}$ = 0.0411, *V*_D_ = 1.8249, *L* = 1.5508, *P *= 0.0411) indicated LD because *V*_D_ > *L* [40].

**Fig. 2:**
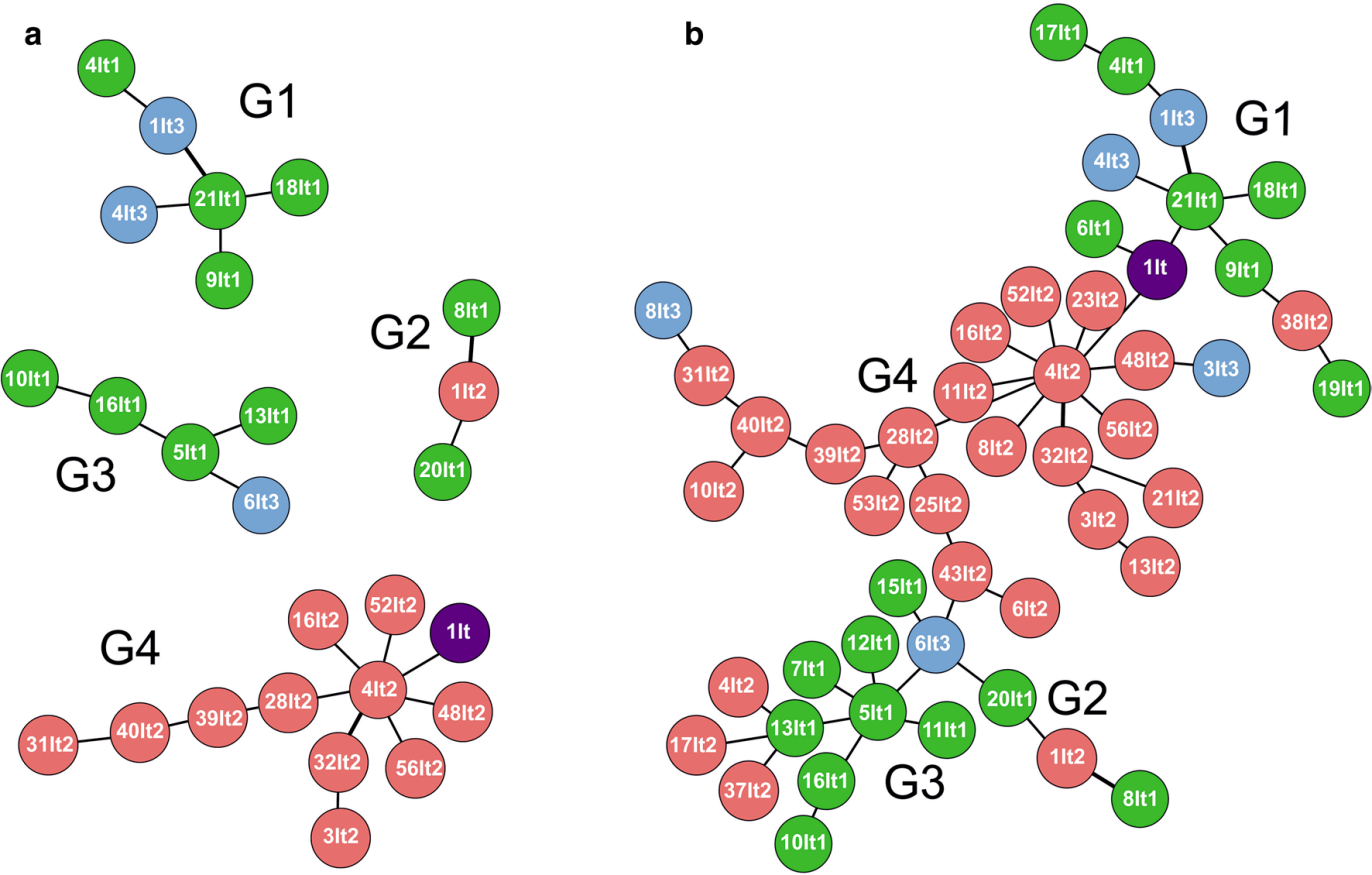
eBURST analysis of 49 northern Italian *Neospora caninum* samples. Specimens from Lombardy (red dots) tend to cluster separately from those from Piedmont (green dots) or Veneto-Trento (blue dots). **a** Analysis using the Double Locus Variant (eBURST DLV) option shows that *N. caninum* multilocus genotypes can be separated into four groups (G1–4). Genotyping of an Italian isolate for which the province of origin was not known (violet dot) revealed results close to most isolates from Lombardy (group G4). **b** The full MST option in eBURST, including the complete data set (*n* = 50 *N. caninum* samples) shows the location of the individual groups within a network of all samples. The analysis was restricted to microsatellite markers available from this and a previous study [31]. Only samples that could be typed for all microsatellite markers were included. Moreover, MS10, which combines variation in three separate motifs (sub-loci), was analysed per each motif individually. Groups G1–G3 resemble the grouping reported in [31], while G4 represents a new group including 11 of 25 bovine *N. caninum* samples from Lombardy that were added to the analysis by this study

Network analysis using eBURST with double locus variation (DLV) among six loci and three MS10 motifs or sub-loci (MS10.1-3) revealed that many *N. caninum* MLMGs from Lombardy (*n* = 11/25; this study) clustered separately (eBURST G4) from those obtained from Piedmont (*n* = 10/17, eBURST G1, G2, G3) or Veneto-Trento (*n* = 3/6, eBURST G1, G2) (Fig. 2a). The grouping of isolates from Piedmont and Veneto-Trento had already been assessed by others [31]. This previous grouping of particular *N. caninum* isolates into the eBURST groups G1, G2, and G3 matched perfectly (*n* = 10/10) with our grouping (Additional file 1: Table S1). Genotyping of an Italian isolate, for which the province of origin was not known (violet dot), revealed results close to most isolates from Lombardy (group G4). The full representation of the MLMST network using the MST option in eBURST, including the complete data set (*n* = 49 *N. caninum* samples), shows the G4 samples in the centre with most of the Veneto-Trento samples on the right side (G1) and the Piedmont samples both left (G2, G3) and right (G1) in the network (Fig. 2b).

**Fig. 3:**
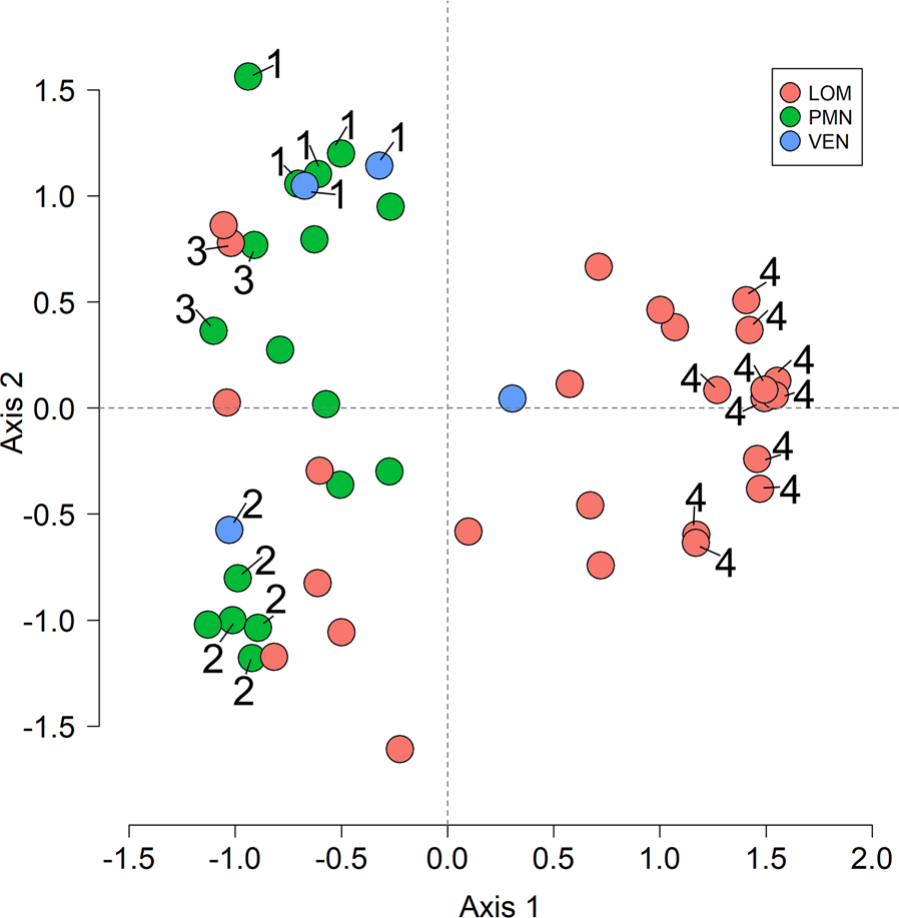
Principle coordinate analysis (PCoA) separates north Italian *N. caninum* based on their MLMGs into clusters. Data from the present and a previous study conducted in cattle from northern Italy [31] were used for this analysis. The two dominant eigenvalues of a PCoA based on the allele-sharing coefficient separated all *N. caninum*-positive samples isolated from Piedmont (PMN) and most from Veneto-Trento (VEN), so that they clustered on the left side of the PCoA graph, while most of the samples from Lombardy (LOM) were located on the right side of the PCoA graph. Groups G1–4 (represented by circles with numbers 1–4), as determined by eBURST, were located clearly in separate positions. While G1–3 was found on the left side, G4 (exclusively Lombardy samples) was localised on the right side of the PCoA graph

Investigating *N. caninum*-positive samples (*n* = 48, excluding a sample for which the province of origin was unknown and the goat sample) by PCoA revealed a clear Axis 1 separation of the samples from Piedmont (Fig. 3) and Lombardy (Fig. 3). While *n* = 17/17 samples from Piedmont were located on the left side, all from Lombardy (*n* = 17/25) were on the right side (Fig. 3). The majority (*n* = 3/5) of samples from Veneto-Trento (Fig. 3) were left or right in the PCoA graph.

When the eBURST grouping (DLV option) was compared to the PCoA result, eBURST G1 and eBURST G3 were located separately in the upper left quarter of the PCoA graph, while eBURST G2 was located in the lower left part (Fig. 3). *Neospora caninum* samples that had been separated by eBURST into G4 (exclusively Lombardy samples) clustered in both the upper and lower corners on the right side of the PCoA graph (Fig. 3).

Differences between the *N. caninum* populations in northern Italy were assessed using *F*_ST_ analysis values (*F*) and Nei’s unbiased genetic distance (*D*) (Table 5). Both results for *F* and *D* suggest that there is a statistically significant genetic difference between *N. caninum* from Piedmont and Lombardy as well as between Lombardy and Veneto-Trento (Table 5)*.*

**Table 5:** Genetic differences between northern Italian *N. caninum*

		*F*_ST_ analysis values (*F*_ST_)		
	Region	Piedmont	Lombardy	Veneto-Trento
Nei’s genetic distance (D)	Piedmont	NA	0.1559**	0.0279
	Lombardy	0.1561**	NA	0.0973*
	Veneto-Trento	0.0331	0.1082*	NA

Pairwise population matrix of *F*_ST_ analysis values (*F*) and Nei’s unbiased genetic distance (*D*). Values written in italics indicate statistical significance based on the goodness-of-fit test (*G*-test) assessing the significance of the effect of region on genetic differentiation

^*^P = 0.012; ^**^P = 0.001

**Fig. 4:**
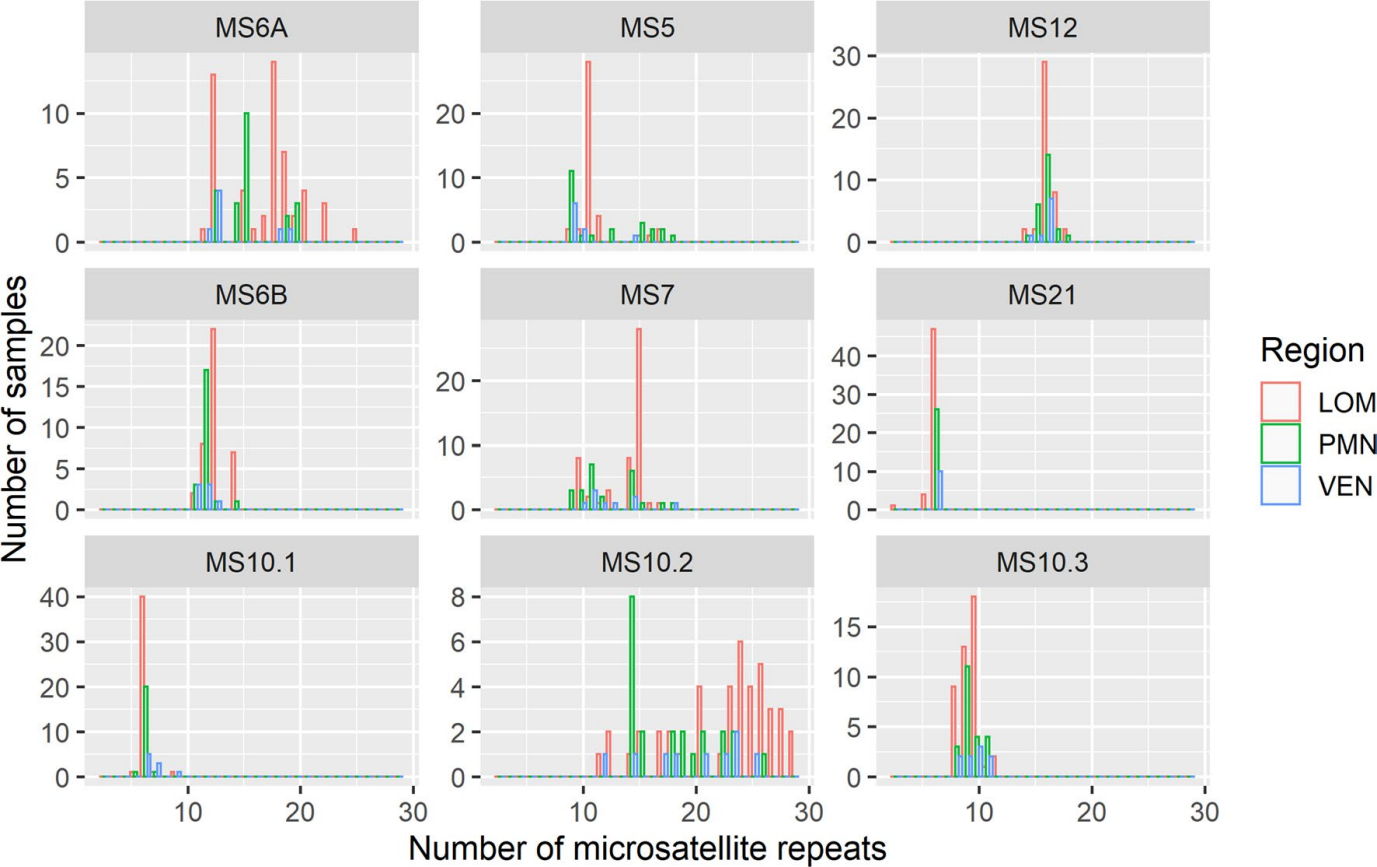
Differences in the heterogeneity of the numbers of repeat units characteristic for *Neospora caninum* microsatellite loci examined in the present and a previous study conducted in cattle from northern Italy [31], i.e. in the regions Lombardy (LOM), Piedmont (PMN), and Veneto-Trento (VEN). The range between the lowest and highest number of repeats was largest for MS10.2 (17 repeats), followed by MS6A (13), MS5 (9) and MS7 (9). For the remaining repeats, the range was four (MS12, MS21, MS10.1, MS10.3) or three repeats (MS6B). Pairwise Wilcoxon test (BH adjusted *P* value) revealed statistically significant differences in MS5 (LOM *vs* VEN), MS6B (LOM *vs* PMN, LOM *vs* VEN), MS7 (LOM *vs* PMN), MS10.1 (LOM *vs* VEN, PMN *vs* VEN) and MS10.2 (LOM *vs* PMN, LOM *vs* VEN)

The graphical representation of the heterogeneity in numbers of repeat units, stratified for various loci, suggested differences between bovine *N. caninum* sampled in the North Italian regions of Lombardy, Piedmont, and Veneto-Trento (Fig. 4). The range between the lowest and highest number of repeats was largest for MS10.2, followed by MS6A, MS5, and MS7. However, the pairwise Wilcoxon test (BH adjusted *P* value) revealed statistically significant differences between regions only for MS5, MS6B, MS7, MS10.1, and MS10.2 (Fig. 4).

## Discussion

This study focused on microsatellite multilocus genotyping of *N. caninum* in bovine foetuses sampled in Lombardy, northern Italy, between 2015 and early 2019 from dairy herds suffering from reproductive problems. The farms selected for this study had a background of *N. caninum* infections in the past as the results of serological testing of a limited number of cattle per herd had indicated; 27.8% of aborted foetuses (55/198) sampled in 43/165 farms tested positive for *N. caninum,* which shows that this parasite is present in many herds of this region. Because pooled samples of brain, lung, and liver had been used to streamline the analytic process, the diagnostic sensitivity might have been reduced and the analysis of individual and further organs (including also cotyledons) could have increased the proportion of positive findings. Since it was not the aim of this study to assess the importance of *N. caninum* as a cause of bovine abortion, we did not collect information on differential diagnoses, i.e. on potential other infectious or non-infectious causes of bovine abortion. It is therefore possible that not all *N. caninum*-positive abortions observed herein were caused by neosporosis. In addition to PCR, further diagnostic steps would have been necessary to confirm neosporosis, including histological and serological examinations as well as the careful exclusion of other causes of abortion [44].

A similar study had also focused on MLMGs in *N. caninum* in cattle and a goat from northern Italy [31]. Both beef (Piedmontese breed) and dairy (Italian Holstein Friesian breed) as well as crossbreeds or local cattle breeds were included, whereas our work was focused on Italian Holstein Friesian cattle under intensive farming conditions. Regarding the geographical area, this previous study had concentrated on bovine foetuses from the regions of Piedmont and Veneto-Trento (*n* = 38) and included only a few samples (*n* = 2) from Lombardy. In addition, the previous study lacked detailed data on the geographic origin of samples. The regions of Piedmont and Veneto-Trento neighbour our study area; Piedmont is located west and Veneto-Trento east from Lombardy. On the basis of microsatellite typing results, it has been hypothesized that local *N. caninum* subpopulations exist in Europe [30,32].

Entirely unexpected was our finding that geographic distance in such a small area as Lombardy (< 30,000 km²) may at least partially explain the MLMG-based genetic distance of *N. caninum* isolates. This suggests that MLMGs remain relatively constant over time in particular herds and only slowly disperse in an area, probably due to animal trade. As it can be assumed that trade between herds was mostly local, this may have resulted in the observed correlation of the geographic distance with the MLMG-based genetic distance between *N. caninum* isolates. Within herds, *N. caninum* seems to be transmitted endogenously mainly, i.e. from latently infected dams to their offspring. However, even an exogenous transmission via oocysts shedding definitive hosts (e.g. farm dogs or wolves) could explain the conserved microsatellite patterns we observed, if the definitive hosts were infected only with a single strain, i.e. the local one, which results in a so-called uniparental mating of *N. caninum* [11]. Both endogenous and exogenous transmission without or only limited contact to neighbouring herds or infectious material might have resulted in local *N. caninum* sub-populations.

All cattle analysed in our study in Lombardy belonged to the Italian Holstein Friesian breed, which was founded in the late twentieth century by the importation of cattle from The Netherlands and North America [45,46]. This breed originates from the north of The Netherlands and Germany and became the predominant dairy breed world-wide; present Holstein Friesian cattle represent crosses of the Dutch Friesian and North-American Holstein lines [45,46]. It has been hypothesized that individual *N. caninum* strains may have been imported together with the Holstein Friesian cattle breed into Italy [31]. Although MLMGs of *N. caninum* from northern Italy showed a relationship to those from Germany [31], it is almost impossible to clarify, whether the *N. caninum* genotypes now observed in Italian Holstein Friesian cattle originated from Italy (i.e. from local cattle breeds) or were introduced by the importation of cattle from abroad. Attempts to find genetic differences between *N. caninum* isolates from Holstein Friesian and from local breeds (i.e. the Piedmontese breed), which could have supported the hypothesis of importation, failed [31]. Interestingly, it has been observed that Argentinian *N. caninum* isolates are still related to those from Spain, even long after the importation of the first Iberian cattle into South America in the fifteenth century [28].

In a few herds, we were able to sample more than a single foetus, and in four of these herds, several months or even a period of years separated the sampling dates. In contrast to findings in bovine herds with epidemic abortions (i.e. in herds where a point source exposure to *N. caninum* oocysts shed by a dog was assumed [10]), particular microsatellite loci differed strongly (i.e. by 3 or even more repeats). It may be discussed whether these larger differences in single loci (MS7, MS6A) or sub-loci (MS10.2) might be an indication for sexual recombination in a definitive host (probably by a farm dog) in the past. However, typing details (Table 2) revealed that related loci on the same chromosome (like MS1B, MS6B) or even sub-loci (e.g. MS10.1 or MS10.2) were not affected. Thus, these differences are best explained by the loss or gain of repeat units, which is typical for microsatellites and caused by point or polymerase template-slippage mutations [47,48]. Nevertheless, sexual recombination events in these herds cannot be completely ruled out, if this recombination occurred with two or more separate strains (i.e. multi-parental) with very similar microsatellite patterns, e.g. the microsatellite pattern of the local *N. caninum* population.

Our findings in individual herds with more than one *N. caninum* MLMG, but also the overall analysis of the *N. caninum* MLMGs in northern Italy, suggest that particular microsatellite loci may be more prone to variation than others. In our study, especially the sub-locus MS10.2, followed by the loci MS6A, MS5 and MS7, showed the strongest variation in the number of repeats. It is well known that several factors contribute to these differences among microsatellite loci, including repeat number, the sequence of the repeat motif, the length of the repeat unit, and flanking sequences (reviewed in [49–51]). Thus, high variability in particular microsatellite loci may indicate that these loci are of limited importance for the parasite. On the other hand, the observation of changes in particular microsatellite loci raises questions regarding the possible effects of such variations on the affected parasite.

Microsatellites are highly abundant in the noncoding DNA of all eukaryotic genomes [52] and changes in these loci might be “neutral”, but microsatellites may also be located in coding regions and variation can be associated with an altered phenotype (reviewed [50]). A recent study on different *Plasmodium* spp. affecting humans suggested that a significant proportion (one fifth to one third) of microsatellite-related sequences are related to coding sequences. Based on gene ontology, the respective coding sequences can be involved in molecular functions like binding or in biologic processes such as metabolism or reproduction [53]. Although we are far from understanding the biological relevance of microsatellites for *N. caninum*, it would be intriguing if future studies could comprehensively address the microsatellites of *N. caninum* to gain more knowledge on their function. It has so far not been possible to link particular microsatellite patterns to particular traits, e.g. virulence for foetuses [54]. Nevertheless, virulence differences have been observed among *N. caninum* strains also in its main intermediate host, i.e. cattle [15,17,18,20–23], and it would be fascinating to know to which extent differences in microsatellite loci contribute to virulence. Recently, highly virulent isolates of *N. caninum* were shown to express a subset of particular secreted proteins in more abundance [55]. The reasons for differential expression between strains could at least in part be due to differences in microsatellite loci as shown for other eukaryotic cells and organisms [52].

In *T. gondii*, an apicomplexan parasite closely related to *N. caninum*, the situation is similar. Currently, relatively conserved microsatellite loci are used to differentiate dominant clonal lineages of *T. gondii*, while also a number of other microsatellite loci are known, which were called finger-printing microsatellite loci. They can be used to differentiate strains of a single type on a local level [56].

## Conclusions

Our findings confirm the concept of local *N. caninum* sub-populations. For the first time, we could show a correlation between the genetic distances of *N. caninum* isolates based on MLMGs and the geographic distances of the places, where the isolates had been obtained. Our results confirm that sexual recombination in *N. caninum* is a rare event. Possible reasons for this might be that endogenous vertical transmission is dominating and that the chance for a definitive host to feed on intermediate hosts with a mixed infection of viable and non-related *N. caninum* strains is extremely low. More comprehensive studies on microsatellites in *N. caninum* and related species like *T. gondii* should be undertaken, not only to improve genotyping capabilities, but also to understand the possible function of these regions in the genomes of these important parasites.

## Supplementary Information

**Additional file 1**: **Table S1**. Microsatellite data on *Neospora caninum* from norther Italy, including also data on intermediate host species, region, province, sampling site, sampling date, real-time PCR result, eBURST designation, and reference.

## Data Availability

Data supporting the conclusions of this article are included within the article and its additional files. The raw datasets used and analyzed during the present study are available as Additional file 1: Table S1 or from the corresponding author upon reasonable request.

## Abbreviations

MLMG: Multilocus-microsatellite genotype
MS: Microsatellite
PCoA: Principle coordinate analysis
DLV: Double locus variant
LOM: Lombardy
PMN: Piedmont
VEN: Veneto-Trento
